# Immune-Mediated Mechanisms in Patients Testing Positive for SARS-CoV-2: Protocol for a Multianalysis Study

**DOI:** 10.2196/29892

**Published:** 2022-01-25

**Authors:** Giuseppe Ietto, Lorenzo Mortara, Daniela Dalla Gasperina, Domenico Iovino, Lorenzo Azzi, Andreina Baj, Walter Ageno, Angelo Paolo Genoni, Francesco Acquati, Matteo Gallazzi, Giorgia Spina, Grace Coco, Federica Pierin, Douglas Noonan, Andrea Vigezzi, Elisa Monti, Valentina Iori, Federica Masci, Caterina Franchi, Salomone Di Saverio, Giulio Carcano

**Affiliations:** 1 General Emergency and Transplant Surgery Department University of Insubria Varese Italy; 2 Department of Biotechnology and Life Sciences University of Insubria Varese Italy; 3 Department of Medicine and Surgery University of Insubria Varese Italy; 4 Istituto di Ricovero e Cura a Carattere Scientifico Multi Medica Milan Italy; 5 General and Endocrine Surgery University of Insubria Varese Italy

**Keywords:** SARS-CoV-2, COVID-19, immunomodulation, severe acute respiratory syndrome, mechanism, phenotype, immunology, white blood cell, immune system, monocyte, natural killer cell, blood, infectious disease, immune response, antigen, vaccine, immunity, protection, genetics, epidemiology

## Abstract

**Background:**

The novel coronavirus has a high mortality rate (over 1% for patients older than 50 years). This can only be partially ascribed to other comorbidities. A possible explanation is a factor that assures a prompt response to SARS-CoV-2 in younger people, independent from the novelty of the virus itself. A factor is believed to stimulate the immune system and provide immunity against more antigens. The only external stimulation received by healthy people is vaccination (eg, the diphtheria, tetanus, and pertussis [DTP] vaccine). One hypothesis is that vaccination helps develop specific immunity but generates sprouting immunity against antigens in transit. The underlying immunological phenomena are the “bystander effect” and “trained immunity.” The developed immunity gives protection for years until it naturally fades out. After the fifth decade of life, the immune system is almost incompetent when a viral infection occurs, and thus, at this stage, the novel coronavirus can enter the body and cause acute respiratory distress syndrome.

**Objective:**

The initial aim is to demonstrate that blood monocytes and natural killer cells show overpowering hyperactivity, while CD4+ and CD8+ T cells experience impediments to their defensive functions in patients with severe SARS-CoV-2 infection. The secondary objectives are to correlate clinical data and vaccination history with laboratory immune patterns in order to identify protective factors. Subsequently, we are also interested in characterizing the phenotypes and state of the degree of activation of peripheral blood mononuclear cells, including monocytes, natural killer cells, and CD4+ and CD8+ T cells, in healthy subjects vaccinated with the Pfizer vaccine.

**Methods:**

Data will be collected using the following 3 approaches: (1) an experimental analysis to study the innate immune response and to identify genetic profiles; (2) an epidemiological analysis to identify the patients’ vaccination history; and (3) a clinical analysis to detect the immunological profile.

**Results:**

The protocol was approved by the Ethics Committee on April 16, 2020, and the study started on April 27, 2020. As of February 2021, enrollment has been completed. Immunological analysis is ongoing, and we expect to complete this analysis by December 2022.

**Conclusions:**

We will recognize different populations of patients, each one with a specific immunological pattern in terms of cytokines, soluble factor serum levels, and immune cell activity. Anamnestic data, such as preceding vaccinations and comorbidities, biochemical findings like lymphocyte immunophenotyping, and pre-existing persistent cytomegalovirus infection, allow depicting the risk profile of severe COVID-19. Proof of the roles of these immunological phenomena in the development of COVID-19 can be the basis for the implementation of therapeutic immunomodulatory treatments.

**Trial Registration:**

ClinicalTrials.gov NCT04375176; https://clinicaltrials.gov/ct2/show/NCT04375176

**International Registered Report Identifier (IRRID):**

DERR1-10.2196/29892

## Introduction

### Background and Rationale

Coronaviruses are common human and animal pathogens. During epidemics, they cause up to one-third of community-acquired upper respiratory tract infections in adults and probably play a role in severe respiratory infections in both children and adults. However, coronaviruses are responsible for a limited amount of diagnosed pneumonia and acute severe illness, especially in the younger population, and a coinfection is frequently detected in hospitalized patients. Usually, the infection is responsible for mild-to-moderate symptoms that rapidly and spontaneously resolve [1].

The new strain of coronavirus, SARS-CoV-2, belongs to the beta-coronavirus family, and it shares all community-acquired coronavirus transmission routes and symptoms. However, the transmission rate is significantly higher, with a faster viral spread responsible for the worldwide outbreak.

In fact, it is a novel virus that was most likely recently passed from bats to humans, and consequently, it is almost unknown to the human immune system. Besides, SARS-CoV-2 seems to undergo modification rapidly during its spread, further avoiding immune defenses [2].

A higher mortality rate characterizes the novel coronavirus, and it has reached over 3% [3]. The death rate is over 1% only for patients aged over 50 years, and it is under 0.4% until 40 years of age. No fatalities have been declared among children under 10 years of age. Furthermore, the death rate is almost double for males when compared with females [4].

The mortality rate distribution of infected patients can be only partially explained by other comorbidities, in addition to older age. Patients with no pre-existing conditions have a fatality rate of 0.9% [4], due to the rapid spread of the virus among the population and within the infected patient, leading to quick and extensive lung injury. However, the near absence of severe illness in children and generally in patients younger than 40 years cannot be explained. Children represent the age group most exposed to all community-circulating viruses, including coronaviruses. Among younger patients, the behavior of the novel virus is similar to that of seasonal community-acquired coronaviruses that can cause severe (but rarely lethal) infections such as pneumonia and bronchiolitis. Indeed, an efficient immune response rapidly counteracts viral infection, even as this virus grows rapidly and changes continuously. In other words, infants, children, and young people could be infected by coronaviruses and by SARS-CoV-2 itself, but the infection is rapidly self-limited, and probably, most of them do not display symptoms. In contrast, older patients experience severe lung injury as a consequence of a slow immune response owing to the novelty of SARS-CoV-2. A possible explanation for these phenomena is a prompt response to SARS-CoV-2 in younger people compared with older people, independent of the novelty of the virus itself. The response might not be specific, but it can limit the infection in the infected host. This is similar to Bacillus Calmette-Guérin (BCG) vaccine immunization in mice that induced nonspecific protective effects against other pathogens such as *Staphylococcus aureus*, *Listeria monocytogenes*, *Salmonella typhimurium*, and *Schistosoma mansoni*. Monocytes and natural killer (NK) cells were believed to play key roles in triggering such trained immunity in murine studies [5]. In addition, the enhancement of monocyte and NK cell responses following BCG vaccination has been reported in humans, and this is due to epigenetic modiﬁcation at the H3K4me3 (trimethylation of lysine 4 on histone H3) level [6].

However, several mechanisms of trained immunity remain unclear, because the trained immune memory–like phenomenon appears to be for a few months or at least a few years. This immunity is not persistent but progressively fades out. Decreased immune response in the elderly could be responsible for more severe acute respiratory disease from coronaviruses in general and higher mortality from COVID-19, since SARS-CoV-2 can cause an explosive attack against the respiratory system, which is similar to major trauma and allows the development of acute respiratory distress syndrome (ARDS). No evident differences were found in different age groups among healthy people, which could justify the drop in immunity against coronaviruses, except its natural fadeout. The trained immune response acts from the age of 2 years, when hypothetical stimulation occurs, to the fifth decade because of its slow decrease.

Vaccines provide external stimulations in healthy people that trigger immune system responses (eg, diphtheria, tetanus, and pertussis [DTP] vaccines stimulate the immune system). Specific vaccines trigger specific immune actions against specific infectious agents, but provide sprouting immunity against antigens in transit, such as those of coronaviruses and other community-circulating viruses [6]. The underlying immunological phenomena are “bystander effect” and “trained immunity.”

Children receive vaccines during the first year of life. Consequently, they become protected against not only specific pathogens but also multiple antigens from pathogens in transit. The developed immunity gives protection against multiple viral infections for years until naturally fading out. After the fifth decade, this immunity is slower to be recalled and reactivated. At this point, a viral infection will find the immune system almost incompetent, and the virus can enter the organism and cause extensive damage. Among elderly people, severe pneumonia and ARDS are frequently noted when they have a coronavirus infection, flu, etc. The history of pandemic viruses reveals an unexplained phenomenon, which could support this hypothesis. In fact, the “Spanish flu” showed a higher mortality rate among young people, especially those aged 20 to 45 years, which could be partly explained by their life conditions after the end of the First World War.

This epidemiological distribution of mortality rate remains unique, and it has not been noted in the successive pandemics of the 21st century [7]. A possible explanation for this is the advent of vaccines, with extensive vaccination after the 1930s. The population received vaccines 20 years before, and the Asiatic and Hong Kong pandemics showed the highest mortality rates after the fifth decade of life when the partially specific immunity had faded [7,8].

Additionally, transplant recipients and HIV-infected patients, characterized by a compromised immune system, unexpectedly do not seem to experience the worst complications of SARS-CoV-2 infection. An immune system imbalance could play a pivotal role during the viral infection, limiting destructive consequences of excessive inflammation.

### Pathophysiological Considerations

The immune system monitors tissue homeostasis, eliminates damaged cells, prevents tumor cell development, and protects against pathogens or infectious agents. The first-line defense involves chemical-physical barriers, followed by the action of innate immune cells, such as cytolytic NK cells and phagocytic myeloid cells, including monocytes/macrophages, dendritic cells, and granulocytes. These cells are activated by the recognition of specific molecular profiles shared by different families of pathogens (Toll-like receptor [TLR], nucleotide oligomerization domain–like receptor, and caspase activation and recruitment domain), including bacteria and viruses, and the innate immune system acts quickly (within minutes/hours). On the other hand, the acquired immune system requires from 4 to 8 days to develop a primary response, and it is able to eliminate infectious agents more effectively due to the extremely specific recognition function of lymphocytes.

In addition, a characteristic of the acquired immune response is its ability to generate “immunological memory,” the mechanism through which an organism develops a faster and more effective response against a subsequent exposure to the same infectious agent. The main actors and effector cells of acquired immunity are CD4^+^ T helper (Th) and CD8^+^ T cytotoxic lymphocytes, which are responsible for cellular immunity, and B lymphocytes, which are responsible for humoral immunity. For an effective response in the body, the innate and acquired immune systems cooperate, despite differences in specificity and activation times. However, more recently, it has been highlighted that cells of the innate immune system have a certain type of immunological memory [9-11].

Macrophages are innate immune cells that belong to the phagocyte cell type. They reside in all tissues and are the mature forms of monocytes, which circulate in the blood and continuously migrate to tissues where they differentiate. They live relatively long and perform several functions in the innate immune response and subsequent acquired immune response [9]. They have a defensive role against pathogens, and have roles in tissue homeostasis and the inflammatory process. Macrophages can be classified as classically activated M1 and alternatively activated M2 macrophages based on their distinct functional capacities in response to stimuli from the microenvironment. M1 macrophages are stimulated by bacterial infections and cytokines produced by Th1 lymphocytes (interferon [IFN]γ), and have roles in the defense against pathogens and killing of cancer cells. M2 macrophages are stimulated by cytokines produced by Th2 lymphocytes (interleukin [IL]-4 and IL-13), and promote physiological and tumor angiogenesis, wound repair, and suppression of immune responses [12-14]. The induction of specific macrophage functions is closely related to the surrounding microenvironment, which acts as an internal regulator. This phenomenon, called polarization, derives from cell-cell/cell-molecule interaction, which governs the functionality of macrophages within host tissues.

Moreover, enhanced responses of a type similar to memory responses (bystander effect or trained immunity) have been highlighted by various studies on monocytes and macrophages [9]. The mechanism involves epigenetic modification of these phagocytes, with enhanced responses in terms of increased responses through TLRs and the release of proinflammatory cytokines, particularly following BCG vaccination for up to 1 year postvaccination [15]. There is also epidemiological evidence indicating that other vaccines, such as the smallpox vaccine and DTP vaccine, could have beneficial contrasting effects on other microorganisms [16].

Interestingly, the release of delayed IFNα from inflammatory monocytes/macrophages has been shown to play a key role in a mouse model of SARS-CoV-2 infection with pulmonary hyperinflammation [17]. Only genetic depletion of the IFNα/β receptor protected mice from death. In addition, other cytokines released by inflammatory monocytes, such as IL1β, IL-6 (also confirmed in COVID-19 patients recently [18]), and inducible nitric oxide synthase, appeared important.

Therefore, the M2 phenotype (the type representing repairing or constructing macrophages) appears to be the one required to inhibit a strong M1 proinflammatory response associated with the late severe phase of ARDS. Multiple interactions between monocytes/macrophages and NK cells have been described both in vitro in humans [19] and in vivo in a mouse model [20]. In the case of a melanoma metastasis mouse model, Sommariva et al showed that vaccine stimulation with TLR3 that recognizes viral double-stranded RNA (the agonist that mimics this response is polyinosinic polinosinic polycytidylic acid [poly I:C]) and TLR9 that recognizes bacterial DNA (CpG) was able to trigger bidirectional crosstalk between lung alveolar M1-type macrophages and NK cells [20].

NK cells are innate cells and represent 10% to 20% of circulating lymphocytes, a proportion that can vary with age. They are phenotypically characterized by the expression of the CD56 membrane molecule and the lack of expression of the CD3 molecule. They are a population of cells with cytotoxic activity toward cancer cells or virus-infected cells, in which they induce apoptosis owing to the release of perforins and granzymes contained within them. More recently, however, some trained immunity functions of these cells have been characterized, with memory-like responses [21,22] and regulatory functions toward monocytes [23]. The NK subtype with the CD56^dim^CD16^+^ phenotype, which represents about 90% to 95% of NK cells in peripheral blood, has perforin and granzyme release activity with cytotoxic action. The other subtype with the CD56^bright^CD16^−^ phenotype, which represents 5% to 20% of NK cells in peripheral blood, does not have cytotoxic action, but secretes high levels of cytokines such as IFNγ and tumor necrosis factor (TNF)α. However, it has been shown that this second subgroup when exposed in vitro to IL-2, IL-12, or IL-15 can assume a CD56^dim^CD16^+^ phenotype and therefore have cytotoxic activity. A third subpopulation has been identified in pregnancy at the deciduous level, where mechanisms of tolerance toward the fetus must be activated. During the first trimester of pregnancy, decidual NK (dNK) cells with the CD56^superbright^CD16^−^ phenotype show a high incidence up to 50% among immune cells. dNK cells are poorly cytolytic and exhibit proangiogenic functions by releasing vascular endothelial growth factor, placenta growth factor, and IL-8. In the tumor field, it has been shown that NK cells similar to dNK expand at the tumor site and are involved in the mechanism of tumor angiogenesis associated with the growth of solid tumors [24-28]. Furthermore, recent studies have suggested that peripheral blood CD56^bright^CD16^−^ NK cells may play a role in regulating the immune response as they produce adenosine, an immunosuppressive molecule, and are capable of inhibiting the proliferation of CD4^+^ T cells, with a possible regulatory role in autoimmune diseases [29].

There will be an extension of the “Coronavirus Project” aim to study the cells involved in the immune response in order to understand the consequences of specific vaccine stimulation against SARS-CoV-2, parallel to the standard humoral response.

Approximately 60 subjects (aged ≥18 years) from the health care workforce, for whom the date of the first administration of the Pfizer vaccine is already defined, will be studied at the following 3 time points: T0, prior to or on the day of the first administration of the vaccine; T1, between the 14th day after the first administration and the day of the second administration; and T2, between the 32nd and 35th days after the first administration.

### Objectives

#### Primary Objective

The primary objective of the study is to explore phenotypes and estimate the activity degrees of monocytes, NK cells, and CD4^+^ and CD8^+^ T cells (including the CD4/CD8 T ratio) in COVID-19 patients’ peripheral blood. A dominant phenotype of these cell populations in patients affected by severe pneumonia could represent a possible therapeutic target. In fact, severe pneumonia and ARDS seems to follow excessive and disruptive inflammation sustained by monocytes in peripheral blood and macrophages in the lungs. NK cells play a pivotal role in antiviral responses and in regulating the activity of dendritic cells, monocytes, and macrophages, and this makes them extremely intriguing in this study. Simultaneously, cytokines and other soluble factors will be measured in plasma. Differences are expected among COVID-19 patients with varying disease severities and healthy people.

#### Secondary Objectives

The secondary objectives are to identify protective factors, using anamnestic data, such as preceding vaccinations, comorbidities, clinical presentation of COVID-19 in terms of clinical signs and symptoms, and biochemical findings. The recognition of a population of patients safe from excessive inflammation against SARS-CoV-2 could define a risk score for the severity of COVID-19, which could allow for the best clinical choice tailored to each patient. Moreover, favorable immunological pattern identification could be followed by implementation of prophylactic or therapeutic immunomodulatory treatments.

#### Additional Objective

We were also interested in characterizing the phenotypes and state of the degree of activation of peripheral blood mononuclear cells (PBMCs), including monocytes, NK cells, and CD4^+^ and CD8^+^ T cells, in healthy subjects vaccinated with the Pfizer vaccine.

## Methods

### Trial Design

This will be an investigator-initiated, institution-led, nonpharmacological intervention for patients with COVID-19.

### Study Setting

The study will be conducted in “Ospedale di Circolo – ASST Sette Laghi,” a teaching hospital affiliated to the University of Insubria in Varese, Italy.

### Eligibility Criteria

The inclusion criteria will be checked before inclusion in the study. The inclusion criteria are as follows: age ≥18 years and documented SARS-CoV-2 infection, without sequencing to differentiate viral variants.

The exclusion criteria are as follows: refusal to the sign the agreement (informed consent); inability to sign the agreement; and HIV, hepatitis C virus, or hepatitis B virus (positive to hepatitis B surface antigen) infection.

### Informed Consent

Inclusion will be feasible after patient approval, relative approval, or an emergency consent procedure (according to Italian law). The consent forms are available from the corresponding author on request. Day 0 will be considered the day of enrollment.

### Additional Consent Provisions

Informed consent approval includes agreement for the collection of biological specimens in ancillary studies, which will be stored for a maximum duration of 15 years.

### Outcomes

#### Primary Endpoint

The hypothesis is that monocytes, NK cells, and CD4^+^ and CD8^+^ T cells in patients with severe SARS-CoV-2 infection will show functional impairment. Moreover, innate cells will reveal overpowering hyperactivity, while adaptive T cells will show an impairment of activity that can provoke a pathologic inflammatory response with massive production of proinflammatory cytokines, edema, and pulmonary fibrosis.

#### Secondary Endpoints

The secondary endpoints are to correlate clinical data and vaccination history with laboratory immune patterns to identify protective factors for severe COVID-19 and break new ground for advanced therapeutic strategies.

Identifying differences between patients having mild infection with positive outcomes and patients having ARDS requiring ventilatory assistance could be useful to optimize the therapy and to come up with an immunostimulant therapy that can produce specific immunity and can decrease the hyperactivity of the innate response and thus the inflammatory condition.

### Recruitment

The proposed study aims to analyze patients with confirmed SARS-CoV-2 infection by real-time reverse transcription polymerase chain reaction (RT-PCR) assays on throat swabs in order to identify differences in their immune responses. Patients will be recruited at the Emergency Department of “Ospedale di Circolo – ASST Sette Laghi,” University of Insubria in Varese, Italy.

A control group of healthy people will be required for further comparison of the findings. We will consider healthy people (both males and females; aged ≥18 years) without infection (negative for SARS-CoV-2), without symptoms of infectious diseases (neither chronic nor acute), and without chronic pathologies (N=30).

After the informed consent procedure, the sample will be divided into 4 subgroups according to the severity of clinical impairment, with at least 30 people in each subgroup. The severity of pneumonia cases will be measured with SMART-COP (systolic blood pressure, multilobar chest radiography involvement, albumin level, respiratory rate, tachycardia, confusion, oxygenation, and arterial pH) [30-32]. The following 4 categories of patients positive for SARS-CoV-2 infection will be analyzed: (1) asymptomatic patients (AS19); (2) mildly symptomatic patients with fever, tiredness, dry cough, diarrhea, etc and without full-blown pneumonia (PAU19); (3) patients with a diagnosis of pneumonia with a “low” risk score (SMART-COP) (POL19); and (4) patients with a diagnosis of pneumonia with a “moderate/high” risk score (SMART-COP) (ARD19).

### Participant Timeline

The estimated study duration is 6 months from the first to last patient recruitment. Each patient will remain in the study until discharge or death.

### Sample Size

The sample size has been determined to be 120, with 30 patients in each group (AS19, PAU19, POL19, and ARD19). In addition, a control group of 30 healthy individuals (negative for SARS-CoV-2) is required. From our experience, we presume that with this sample size, we would be able to identify a transparent variation in immune cell counts and serum cytokine levels. This conforms to most of the immunologic studies in the literature. Moreover, with this sampling, we can identify significant differences in the order of 20% to 30% in terms of the phenotype and function of immune cells.

### Data Collection and Management

Data will be collected using the following 3 approaches: (1) an experimental analysis for clusters of patients to study the innate immune response and to identify genetic profiles; (2) an epidemiological analysis to identify patient vaccination history; and (3) a clinical analysis to detect the immunological profile.

### Sample Description and Collection for the Experimental Analysis

For the specific analysis, to study the innate immune response and to identify the genetic profiles, we will analyze NK cells, monocytes, and CD4^+^ and CD8^+^ T cells from peripheral blood in both the healthy group (tested negative for SARS-CoV-2) and sick subgroups (AS19, PAU19, POL19, and ARD19). For each patient, a venous blood sample will be obtained (15 mL in EDTA solution).

### Preparation of Plasma and Isolation of PBMCs From Blood

From different groups of patients, blood samples (10-15 mL) will be drawn in EDTA tubes and centrifuged at 360 *g* for 10 minutes to obtain plasma that will be stored at −80°C for subsequent analysis of cytokines and chemokines of interest by enzyme-linked immunosorbent assay (ELISA) (IL-1β, IL-6, TNFα, IFNα, IL-10, IL-12, CCL2, and CXCL10) at the end of enrollment.

The cell pellets will be brought back to the initial volume with phosphate-buffered saline (PBS) (Euroclone) and diluted 1:1 (v/v) with PBS. It will then be subjected to density gradient stratification with Lymphosep (Biowest) at 500 *g* for 30 minutes at room temperature with no brake. The PBMCs derived from the white ring will be collected, washed twice in PBS, and then used for subsequent experiments using a flow cytometer assay [25]. The in vitro culture using PBMCs can vary from ex vivo 1 day to a few days, and cells will be maintained in RPMI 1640 medium (Euroclone), supplemented with 10% fetal bovine serum (FBS) (Euroclone), 2 mM l-glutamine, and 100 U/mL penicillin and 100 μg/mL streptomycin (both Euroclone), at 37°C and 5% CO_2_.

To perform ex vivo flow cytometry cell phenotype analysis, 2.5×10^5^ fresh or frozen total PBMCs per tube will be stained for 30 minutes at 4°C with monoclonal antibodies (mAbs) (Becton Dickinson [BD]) as follows: CD3-BB700, CD56-APC, CD16-PE-Cy7, CD159a (NKG2A)-BV510, NKG2C-PE, NKGD2-PE, DNAM-1-PE, CD25-PerCP-Cy5.5, CD69-PE, CD279 (PD1)-BUV737, TIGIT-BV786, CD96-BB515, and CD366 (TIM3)-BV421. Samples will be acquired using BD LSR Fortessa. Following the forward/side scatter setting, NK cells will be divided into 2 cell subsets (ie, CD3^−^ and CD56^dim^ CD16^+^ cells [CD56^dim^ NK cells, the major subset, about 90%] and CD3^−^ and CD56^bright^ CD16^−/low^ cells [CD56^bright^ NK cells, the minor subset, about 10%]). Other marker expressions will be evaluated in both subsets of gated cells.

For ex vivo flow cytometry cell evaluation of monocyte phenotypes, 2.5×10^5^ fresh or frozen total PBMCs per tube will be stained for 30 minutes at 4°C with mAbs (BD) as follows: CD45-APC, CD14-FITC, CD16-PE-Cy7, PE-CD209, and PE-CD80. Following the forward/side scatter setting, monocytes will be divided into 3 subsets (ie, CD14^++^ and CD16^−^ cells [the classical subset, about 90%], CD14^++^ and CD16^+^ cells [the intermediate subset, about 10%], and CD14^−/low^ and CD16^++^ cells [the nonclassical subset]) [33].

We will also evaluate phenotypes for CD4^+^ and CD8^+^ T cells and the CD4/CD8 T ratio using 2×10^5^ fresh or frozen total PBMCs, as previously described, with the following mAbs (BD): CD3-PerCP, CD4-APC, CD8-V450, and CD25-PE. We will also assess T regulatory (Treg) cells with CD3-PerCP, CD4-APC, and CD25-PE. Furthermore, we will analyze T-cell responses toward the spike protein using 15-mer peptides (6 nM each, final concentration; 11 amino acids overlapping) of the full-length spike protein (Milteny Biotec). The IFNγ response by spike-specific T cells will be studied by intracellular staining with a fluorescence-activated cell sorter. We will perform cytofluorimetric intracellular staining by using a Cytofix/Cytoperm fixation and permeabilization kit, according to the manufacturer’s protocol (BD). Briefly, 5×10^5^ PBMCs will be first stained with surface mAbs, such as CD3, CD4, and CD8, for 30 minutes at 4°C. Then, cells will be washed and treated with 200 μL of cytofix/cytoperm solution for 30 minutes at 4°C. Then, cells will be washed and incubated with anti-IFNγ mAbs (BD) for an additional 30 minutes. After a further washing step, cells will be analyzed using flow cytofluorimetric assay.

PBMCs will also be stimulated in vitro for 6 h with different types of stimuli, such as lipopolysaccharide (LPS) (Sigma Aldrich) (recognized by TLR4), poly (I:C) (Sigma Aldrich) (recognized by TLR3), poly (I:C) plus IL-2 plus IL-12, and phorbol myristate acetate (Sigma Aldrich) plus ionomycin (Sigma Aldrich) in the presence of monensin (Golgi Stop, BD) and brefeldin A (Golgi Plug, BD). This procedure will allow the detection of cytokine/chemokine production from NK cells, monocytes, and T cells by using a flow cytometry intracellular assay, as described previously [34]. For NK cells, we will measure IFNγ-BV650, TNFα-PE, CCL2-PE, and CXCL10-PE by intracellular staining and CD107a-PE (surface degranulation marker) using flow cytofluorimetric assay (BD). For monocytes, we will investigate IL-6-PE, TNFα-PE, IL-12-PE, and CXCL10-PE (BD) (M1-type proinflammatory markers), and TGFβ-PE, IL-10-PE, and CCL18-PE (BD) (M2-type anti-inflammatory markers), and for CD4^+^ and CD8^+^ T cells, we will assess IFNγ-BV650 (BD) by intracellular staining as above.

Whenever the quantity of PBMCs is sufficient, other functional in vitro tests on NK cells and monocytes will be set up. In particular, NK cells within PBMCs will be studied in a 4-h coculture degranulation flow cytofluorimetric assay using NK cells and erytroleucemic K562 cells through assessment of CD107a-PE or CD107a-FITC NK cell surface staining [35]. Moreover, CD14^+^ monocytes and CD3^−^CD56^+^ NK cells will be purified with specific mAbs linked to microbeads (Miltenyi Biotec) and a magnetic separator to obtain >90% purified cell populations.

Purified monocytes and purified NK cells will be cultured using RPMI 1640 medium with 10% FBS and supplementation with macrophage colony-stimulating factor and IL-2 (both Miltenyi Biotec), respectively. They will be stimulated with different stimuli (see above) and then checked for intracellular cytokines/chemokines of interest (see above). At the same time, monocytes and NK cells can be harvested at the end of the in vitro incubation culture. We will collect conditioned media for cytokine/chemokine detection using ELISA (IL-1β, IL-6, TNFα, IFNα, IL-10, IL-12, and CXCL10) according to the manufacturer’s protocol (R&D Systems).

The 4- to 5-day monocyte culture will be further stimulated for 24 h with LPS (Sigma Aldrich) and IFNγ (Miltenyi Biotec) (M1 stimulus) or IL-4 (Miltenyi Biotec) (M2 stimulus) to investigate macrophage polarization, with assessment of surface M1 markers (TNFα and CXCL10 [BD]) or M2 markers (IL-10 and CCL18 [BD]) to check the prevalence of macrophage polarization in different groups of COVID-19 patients.

The epidemiological analysis will be carried out by integrating both vaccination history and the daily data collected after hospital admission. Azienda a tutela della salute Insubria archives will provide missing data.

Considering the immunological profile, patients with COVID-19 will undergo routine examinations and the following: lymphocyte immunophenotyping; determination of C3 and C4 complement fraction activity; determination of serum immunoglobulin (IgG, IgM, IgA, and IgE) levels; serum protein electrophoresis; determination of serum angiotensin converting enzyme levels [36,37]; cytomegalovirus serology tests; and determination of serum IL-6 levels.

A specific data collection form will store information quickly, and then, data will be collected in a database.

### Data Management

Data will be collected in the Emergency and Trauma Research Centre, University of Insubria, Varese, Italy by clinical data technicians on an electronic case report form, using double password-protected computers. Prespecified lists, ranges of values, and drop-down menus in the electronic case report form will facilitate data entry and prevent writing errors. Study documents will be deidentified and stored in the Emergency and Trauma Research Centre, University of Insubria, Varese, Italy. All personnel involved in data analysis will be masked. Only the principal investigators and statisticians will have access to the final data set.

### Confidentiality

People with direct access to the data will take all necessary precautions to maintain confidentiality. All data collected during the study will be rendered anonymous. Only initials and inclusion numbers will be registered.

### Plans for the Collection, Laboratory Evaluation, and Storage of Biological Specimens

On day 0, blood samples (15 mL) will be drawn in EDTA tubes, centrifuged, and stored (−80°C) for subsequent analysis. After the first analysis, the remaining biological specimens will be kept for a maximum duration of 15 years.

### Statistical Methods for the Primary and Secondary Outcomes

Associations between different variables will be examined using Pearson correlation coefficients. Paired comparisons will be made using the Wilcoxon matched pairs test. Nonpaired comparisons will be made using the 2-tailed Mann-Whitney test. Multiple comparisons will be made using appropriate one-way analysis of variance (ANOVA). A *P* value <.05 will be considered statistically significant. All statistical analyses will be performed using R version 3.4.3 (R Project for Statistical Computing) and SPSS 21 (SPSS Inc).

### Plan for Access to the Full Protocol, Participant-Level Data, and Statistical Code

The protocol is available on the ClinicalTrials.gov website (NCT04375176). Study documents will be deidentified, stored by the Emergency and Trauma Research Centre, University of Insubria, and kept for at least 15 years in a locked secure office. All personnel involved in data analysis will be masked. Only the principal investigators and statisticians will have access to the final data set.

### Oversight and Monitoring

#### Composition of the Coordinating Center and Trial Steering Committee

The coordinating center is the Emergency and Trauma Research Centre, University of Insubria, Varese, Italy. The steering committee includes GI, G Carcano, and LM. The data management team includes DI and DDG.

### Dissemination Plans

The results of the study will be released to participating physicians, referring physicians, and the medical community no later than 1 year after the completion of the trial, through presentations at scientific conferences and publications in peer-reviewed journals. Eligible authors will meet all 4 requirements of the ICMJE guidelines: (1) Substantial contributions to the conception or design of the work; or the acquisition, analysis, or interpretation of data for the work; (2) Drafting the work or revising it critically for important intellectual content; (3) Final approval of the version to be published; (4) Agreement to be accountable for all aspects of the work in ensuring that questions related to the accuracy or integrity of any part of the work are appropriately investigated and resolved.

### Ethics Approval

Protocol version 1.0 was approved by the Ethics Committee on April 16, 2020. The clinical trial will adhere to the principles of the Declaration of Helsinki and to the Clinical Trials Directive 2001/20/EC of the European Parliament on the approximation of the laws, regulations, and administrative provisions of the Member States relating to the implementation of Good Clinical Practices in the conduct of clinical trials on medicinal products for human use. All trial-related activities will be conducted with written informed consent and in accordance with relevant local and national guidelines.

## Results

 The study started on April 27, 2020. As of December 2020, we have enrolled 120 patients who have tested positive for SARS-CoV-2 and 30 healthy subjects. During January and February 2021, we enrolled 60 subjects from the health care workforce after they received the Pfizer vaccine.

The clinical data set is complete. Immunological analysis is ongoing, and we expect to complete this analysis by December 2022.

## Discussion

According to the medical hypothesis on which the protocol is based, young people could benefit from functional adaptation of innate immune cells induced through epigenetic reprogramming and, especially, pre-existing “partially specific” immunity to community viruses associated with the “bystander effect” of preceding vaccinations [6]. In this study, we will explore the main differences among patients infected by SARS-CoV-2 who experience the illness at different degrees of severity. We will recognize different populations of patients, each one with a specific immunological pattern. There could be differences in terms of cytokines, soluble factor serum levels, and immune cell activity (both of the innate compartment and the acquired one). In this way, the contribution of trained immunity and the bystander effect to protection against SARS-CoV-2 could be explained. Anamnestic data, such as preceding vaccinations and comorbidities, biochemical findings like lymphocyte immunophenotyping, and pre-existing persistent cytomegalovirus infection, allow depicting the risk profile of severe COVID-19. Proof of the roles of these immunological phenomena in the pathogenesis of COVID-19 can be the basis for the implementation of therapeutic immunomodulatory treatments. In addition, the definition of an immunological risk profile could tailor established therapies to each type of patient.

## Abbreviations

ARDS: acute respiratory distress syndrome
BCG: Bacillus Calmette-Guérin
BD: Becton Dickinson
dNK: decidual natural killer
DTP: diphtheria, tetanus, and pertussis
ELISA: enzyme-linked immunosorbent assay
FBS: fetal bovine serum
IFN: interferon
IL: interleukin
LPS: lipopolysaccharide
mAb: monoclonal antibody
NK: natural killer
PBMC: peripheral blood mononuclear cell
PBS: phosphate-buffered saline
RT-PCR: reverse transcription polymerase chain reaction
SMART-COP: systolic blood pressure, multilobar chest radiography involvement, albumin level, respiratory rate, tachycardia, confusion, oxygenation, and arterial pH
Th: T helper
TLR: Toll-like receptor
TNF: tumor necrosis factor
